# Supplementary material for: High figure-of-merit for ZnO nanostructures by interfacing lowly-oxidized graphene quantum dots
Source: Nat Commun. 2024 Mar 14;15:1996. doi: 10.1038/s41467-024-46182-2 (PMC10940299; doi:10.1038/s41467-024-46182-2)
Supplement: Supplementary file 1 — Supplementary Information [file 41467_2024_46182_MOESM1_ESM.pdf]

Supplementary Information

High Figure-of-merit for ZnO Nanostructures by Interfacing Lowly-Oxidized Graphene Quantum Dots

Choi et al.

Table of Contents

Supplementary Figure 1| Fabrication procedure of 3D thin-shell ZnO.

Supplementary Figure 2| Optimization of the shell thickness of 3D ZnO in terms of thermal conductivity and structural stability.

Supplementary Figure 3| X-ray diffraction (XRD) spectra of 3D ZnO with varying grain sizes.

Supplementary Figure 4| Scanning electron microscopy (SEM) images of the transferred 3D ZnO film onto a microfabricated thermoelectric measurement platform (MTMP).

Supplementary Figure 5| Atomic force microscopy (AFM) image of dispersed graphene quantum dots (GQDs) on SiO_2/Si.

Supplementary Figure 6| Chemical and compositional analysis of 3D GQD@ZnO.

Supplementary Figure 7| X-ray photoelectron spectroscopy (XPS) analysis of GQDs.

Supplementary Figure 8| XPS analysis of 3D GQD@ZnO.

Supplementary Figure 9| UV-vis absorbance spectra of 3D GQD@ZnO, 3D ZnO, GQDs, and ZnO thin film.

Supplementary Figure 10| Photographs of GQDs solution with varying GQD concentration.

Supplementary Figure 11| Hall measurement results of 3D GQD@ZnO.

Supplementary Figure 12| Temperature-dependent weighted mobility of 3D ZnO (small grain) and 3D GQDx@ZnO (x = 1, 2, 3, and 4).

Supplementary Figure 13| Thermal stability of GQDs.

Supplementary Figure 14| Five sets of measurements for the thermoelectric properties of 3D GQD@ZnO.

Supplementary Figure 15| Estimated thermal conductivity and zT values at 580 K for 3D GQDx@ZnO (x = 3) as a function of grain size for ZnO.

Supplementary Figure 16| Phonon mean free path (MFP) for bulk ZnO, 3D ZnO with different grain sizes, and 3D GQDx@ZnO (x = 1, 2, 3, and 4) at 300 K.

Supplementary Figure 17| XRD spectra of 3D ZnO and 3D GQDx@ZnO (x = 1, 2, 3, and 4).

Supplementary Figure 18| Measurement system of electrical conductivity for 3D GQD@ZnO.

Supplementary Table 1| Thermoelectric properties of various metal oxides in previous studies.

Supplementary Table 2| Thermoelectric parameters of 3D ZnO with varying grain sizes.

Supplementary Table 3| Thermoelectric parameters of 3D GQDx@ZnO (x = 1, 2, 3, and 4).

Supplementary Table 4| Materials parameters used to calculate the lattice thermal conductivity.

Supplementary References

Supplementary Figures

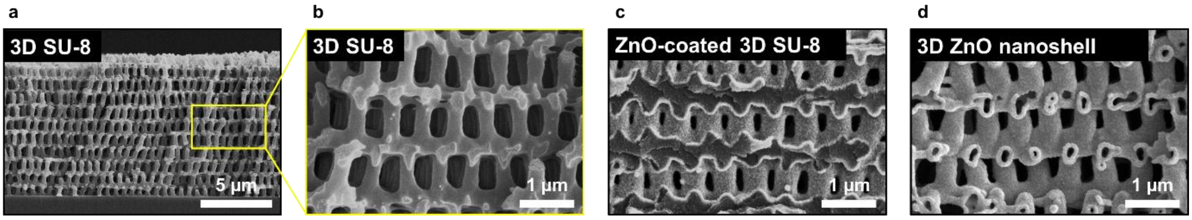

Supplementary Figure 1| Fabrication procedure of 3D thin-shell ZnO. a, Scanning electron microscopy (SEM) image of 3D SU-8 nanostructure polymeric template. **b,** Magnified SEM image of **(a)**. **c,** SEM image of ZnO-coated 3D SU8 nanostructure polymeric template. **d,** SEM image of the 3D ZnO thin-shell after etching the SU-8 polymeric template.

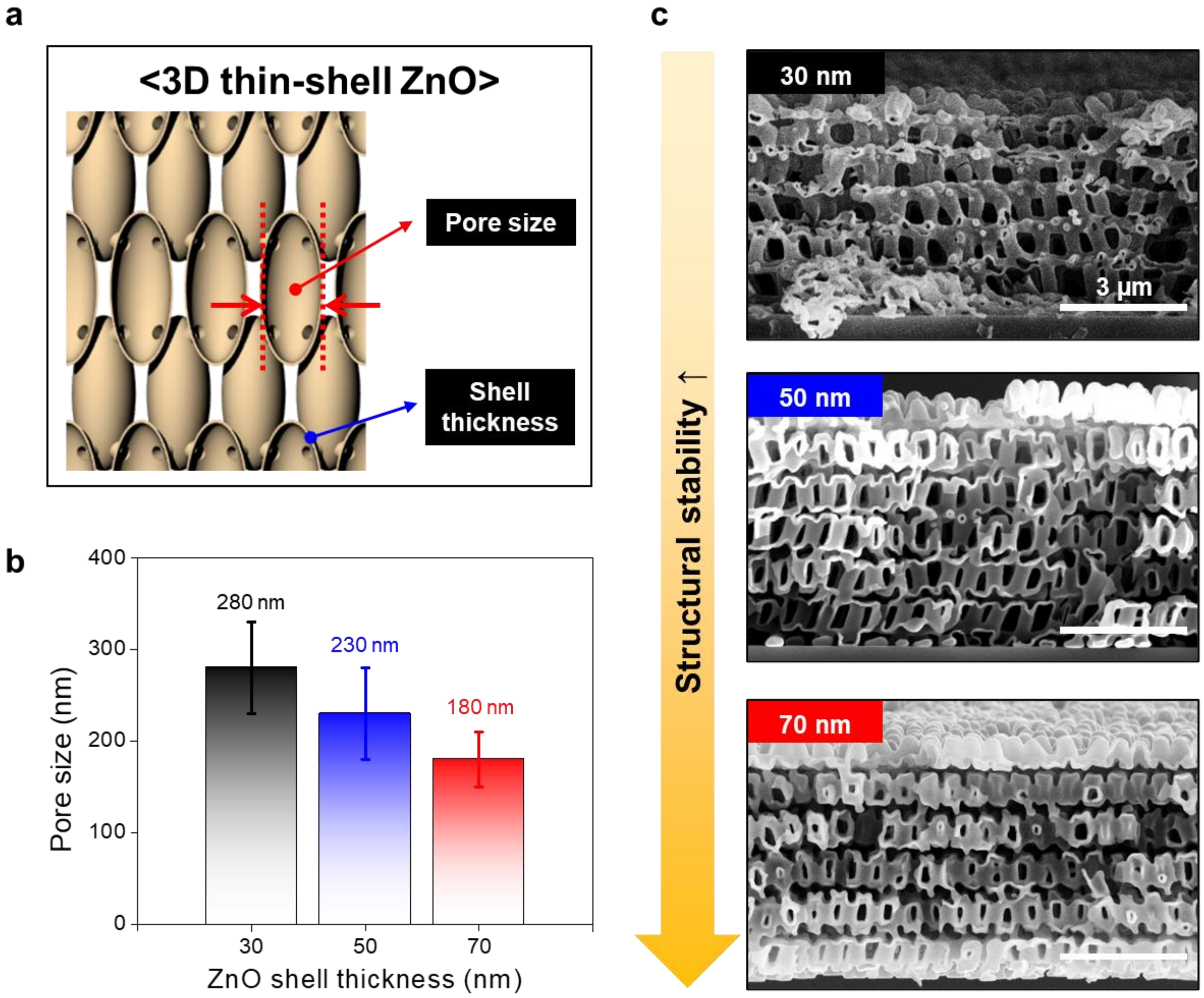

Supplementary Figure 2| Optimization of the shell thickness of 3D ZnO in terms of thermal conductivity and structural stability. a, Schematic illustration of 3D thin-shell ZnO. **b,** Pore sizes of 3D ZnO with 30, 50, and 70 nm shell thickness. **c,** Structural stability of 3D ZnO nanostructures according to the shell thickness. Error bars shown in the **Supplementary Fig. 2** represents the standard deviation (SD).

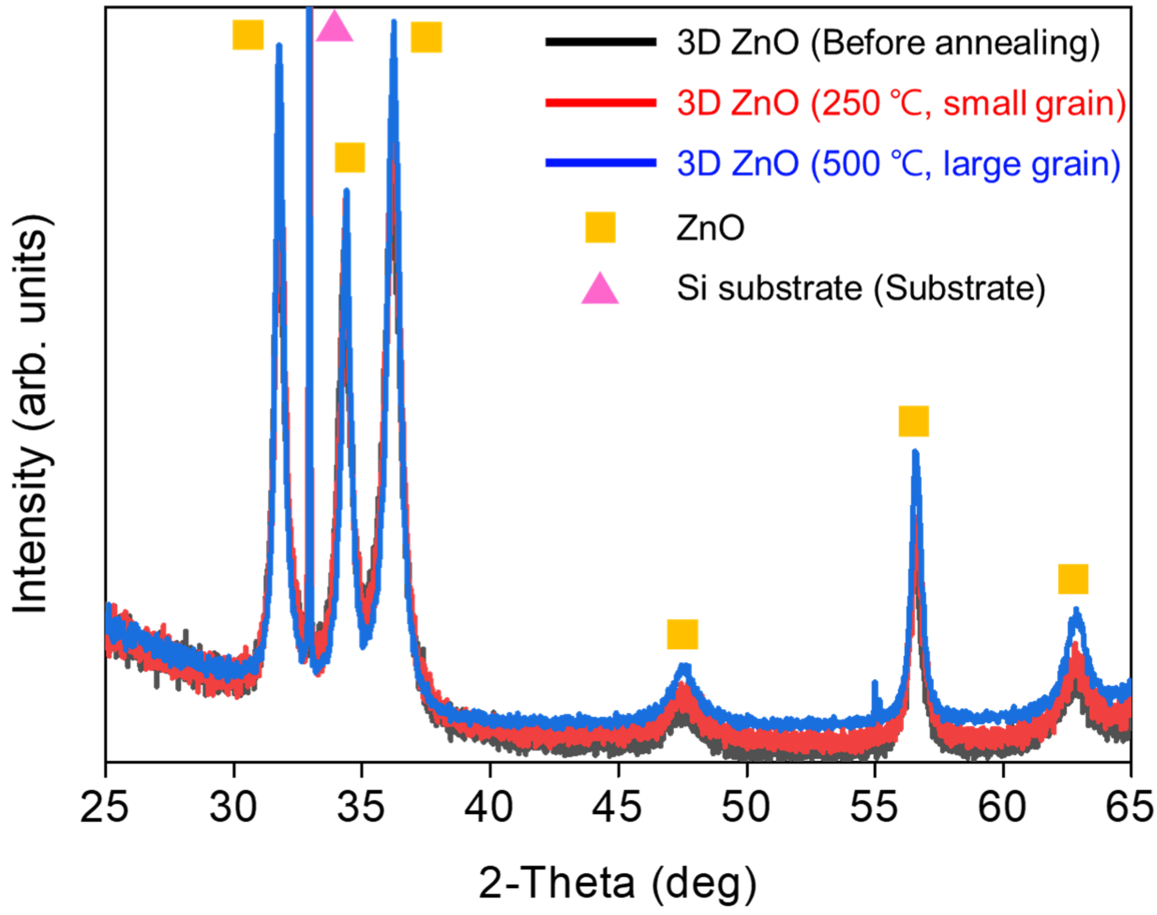

Supplementary Figure 3| X-ray diffraction (XRD) spectra of 3D ZnO with varying grain sizes.

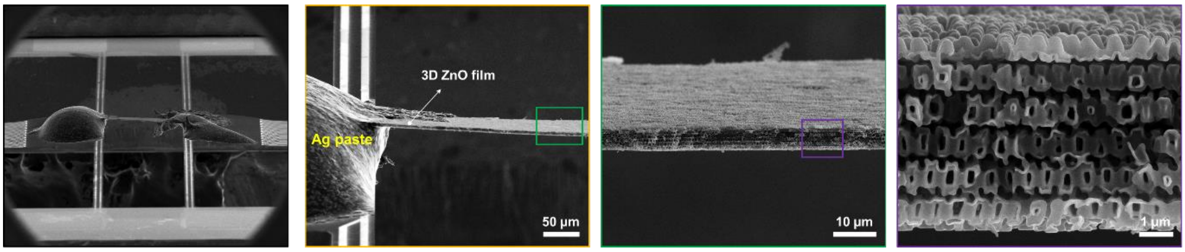

Supplementary Figure 4| SEM images of the transferred 3D ZnO film onto a microfabricated thermoelectric measurement platform (MTMP).

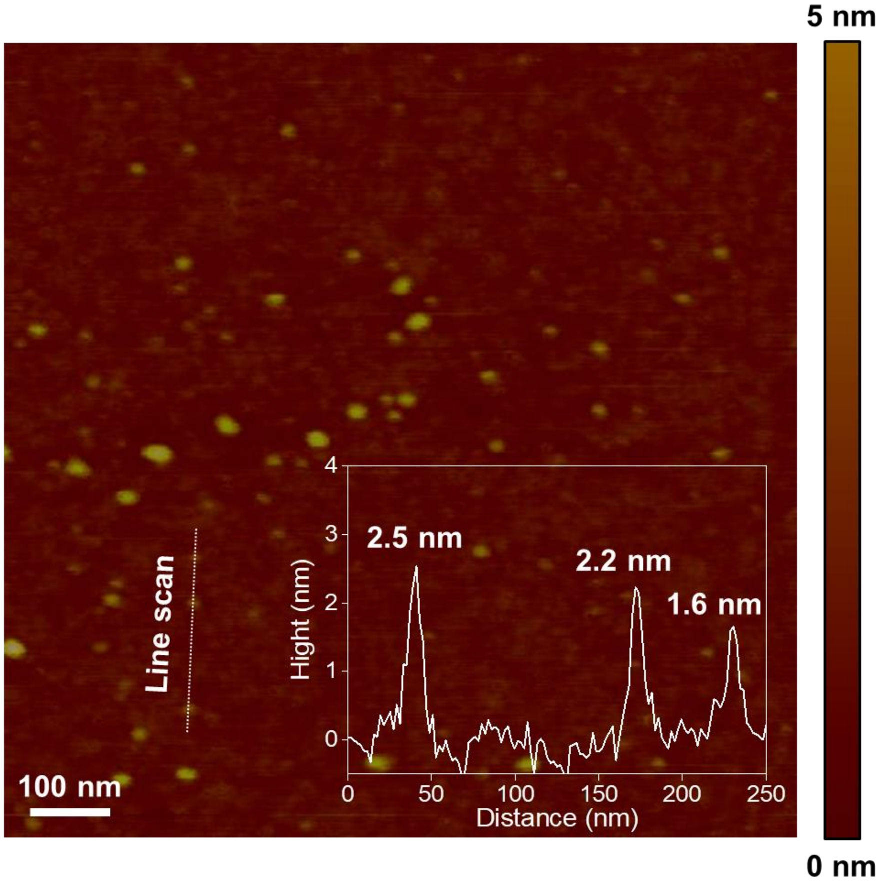

Supplementary Figure 5| Atomic force microscopy (AFM) image of dispersed graphene quantum dots (GQDs) on SiO_2/Si. The inset indicates line scan profile of GQD thickness.

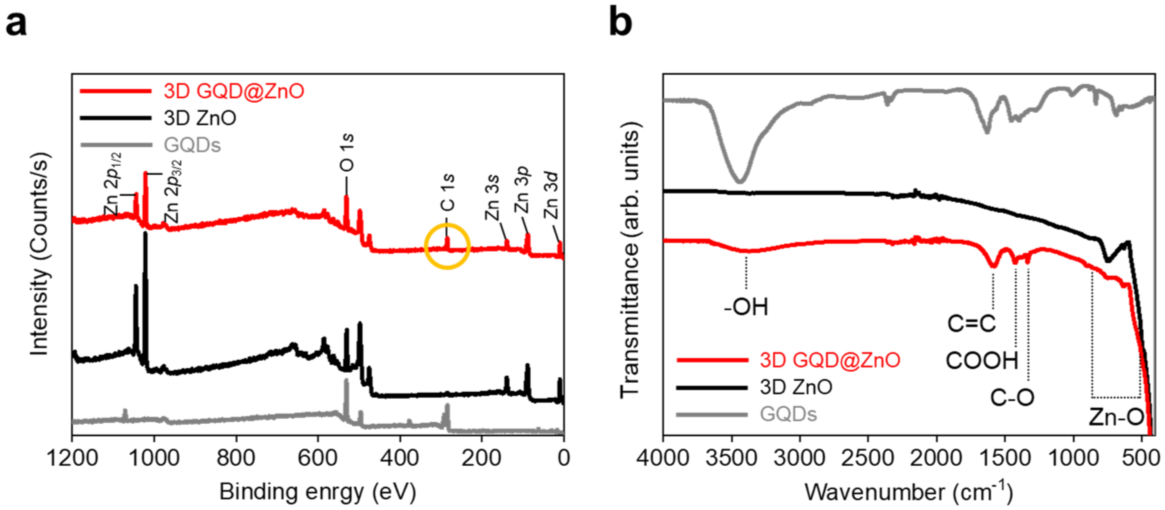

Supplementary Figure 6| Chemical and compositional analysis of 3D GQD@ZnO. a, X-ray photoelectron spectroscopy (XPS) survey results of the 3D GQD@ZnO, 3D ZnO, and GQDs. **b,** Fourier-transform infrared spectroscopy (FTIR) spectra of the 3D GQD@ZnO, 3D ZnO, and GQDs.

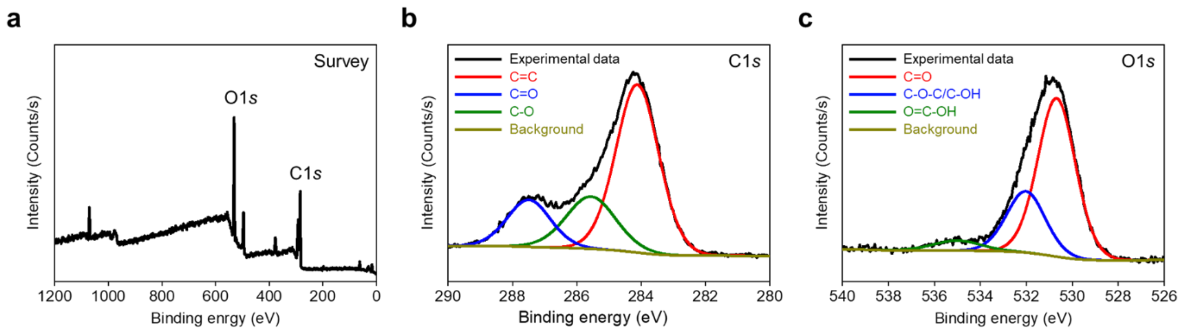

Supplementary Figure 7| XPS analysis of GQDs. a, XPS survey results of the GQDs. **b,** XPS C1*s* peak fitting of the GQDs. **c,** XPS O1*s* peak fitting of the GQDs.

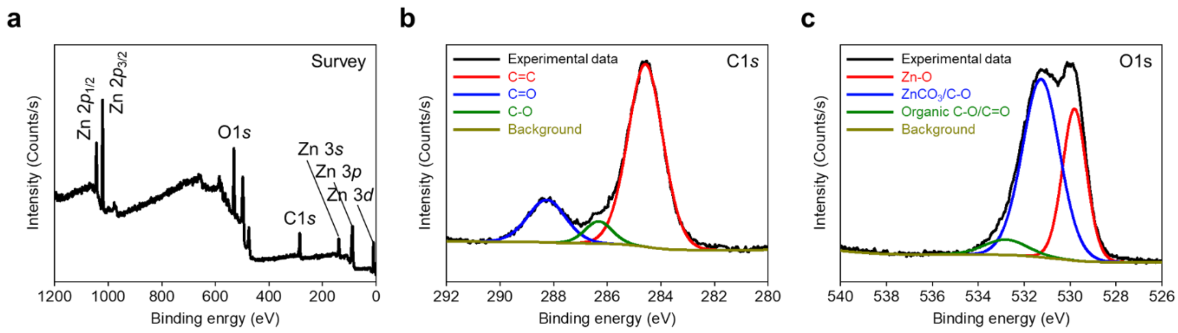

Supplementary Figure 8| XPS analysis of 3D GQD@ZnO. a, XPS survey results of the 3D GQD@ZnO. **b,** XPS C1*s* peak fitting of the 3D GQD@ZnO. **c,** XPS O1*s* peak fitting of the 3D GQD@ZnO.

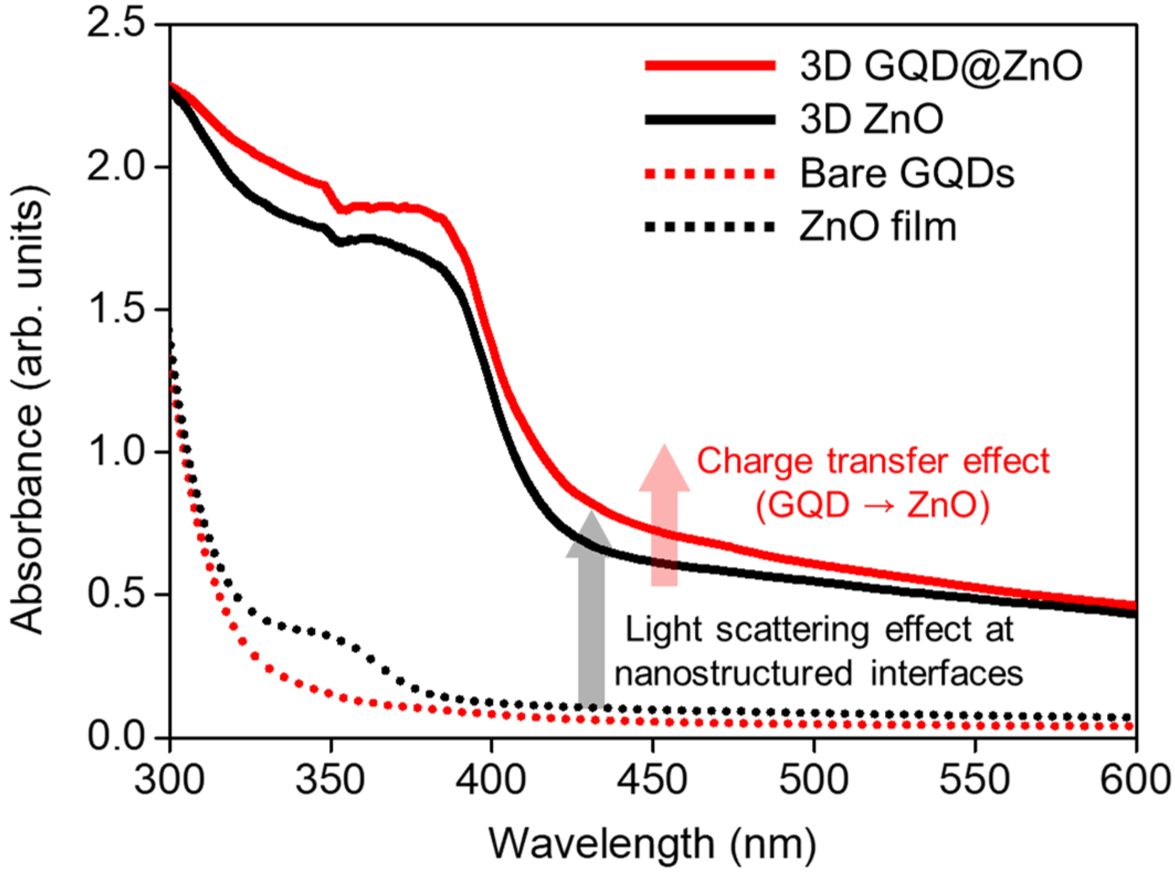

Supplementary Figure 9| UV-Vis absorption spectra of 3D GQD@ZnO, 3D ZnO, GQDs, and ZnO thin film.

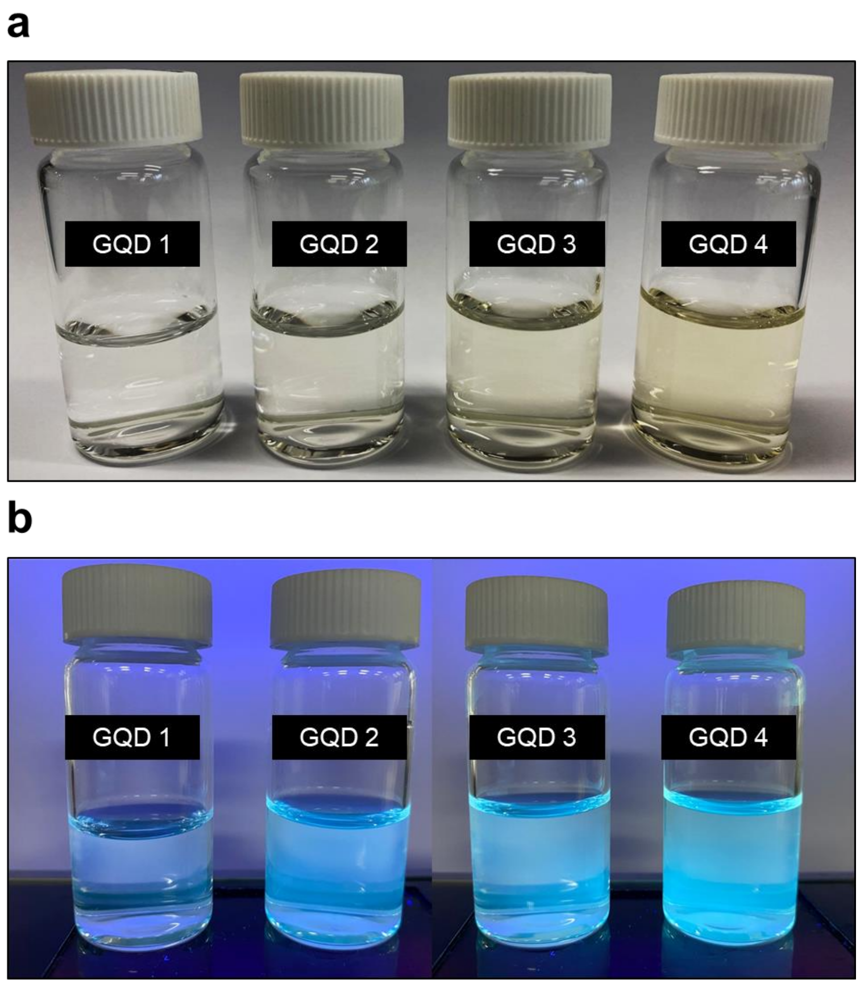

Supplementary Figure 10| Photographs of GQDs solution with varying GQD concentration. a, Image of GQDs solution under ambient light. **b,** Image of GQDs solution under UV light.

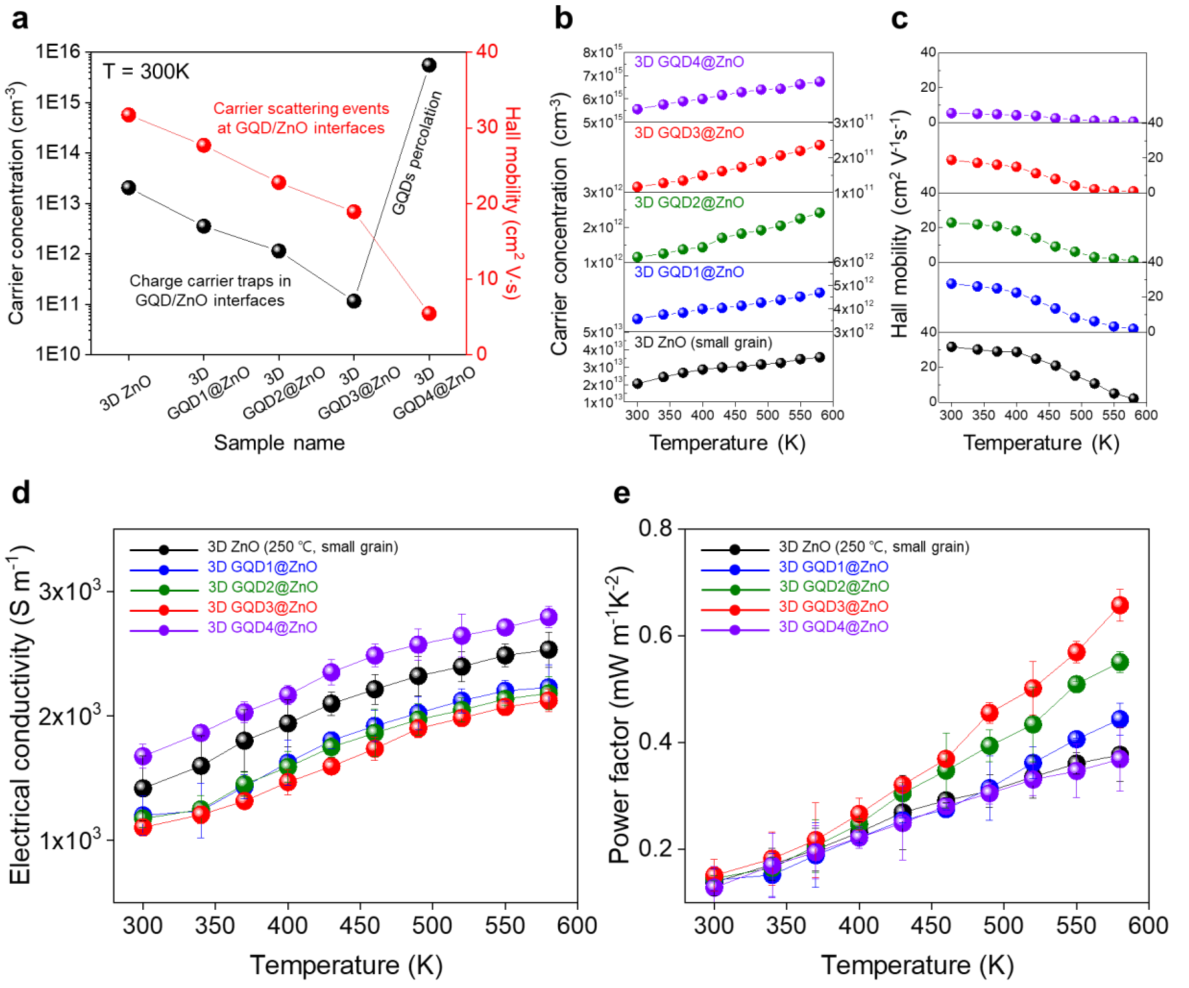

Supplementary Figure 11| Hall measurement results of 3D GQD@ZnO. a, Charge carrier concentration and charge carrier mobility of 3D ZnO (small grain) and 3D GQDx@ZnO (x = 1, 2, 3, and 4) at 300 K. **b,** Temperature-dependent charge carrier concentration and **c**, charge carrier mobility of the 3D ZnO (small grain) and 3D GQDx@ZnO (x = 1, 2, 3, and 4). **d**, Temperature-dependent electrical conductivity and (**e**) power factor for 3D ZnO (small grain) and 3D GQDx@ZnO (x = 1, 2, 3, and 4). Error bars shown in the **Supplementary Fig. 11** represents the SD.

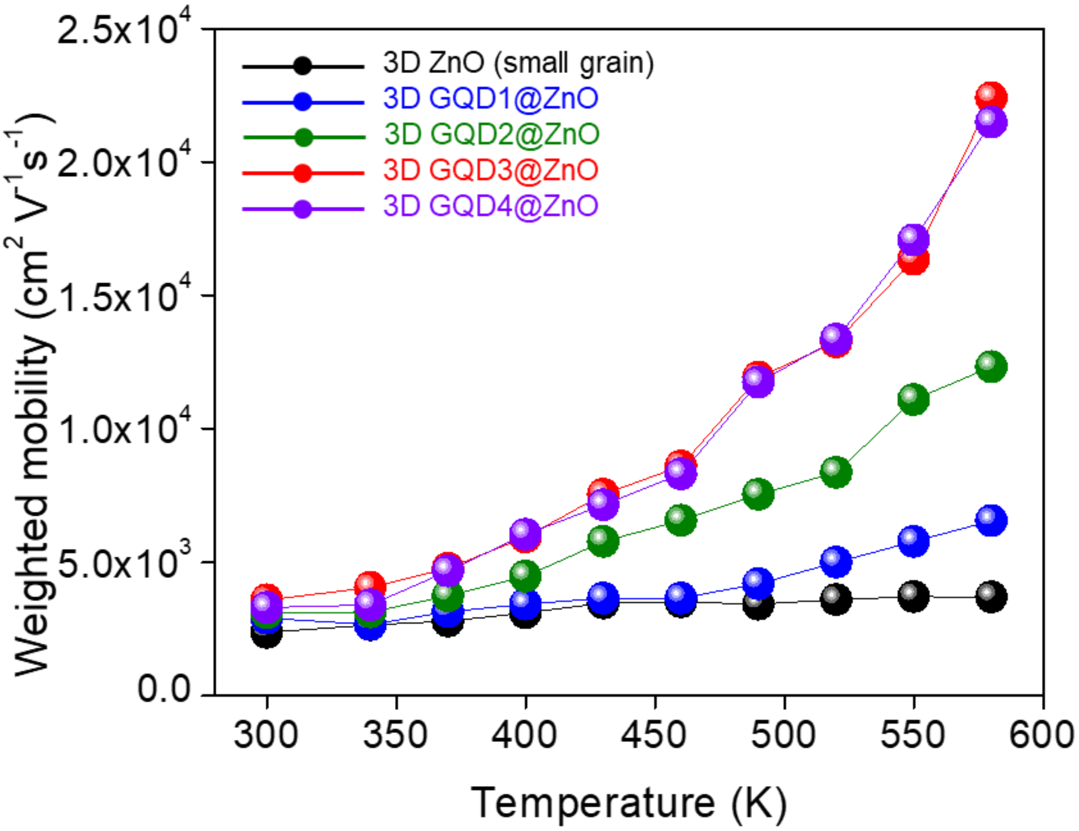

Supplementary Figure 12| Temperature-dependent weighted mobility of 3D ZnO (small grain) and 3D GQD*x*@ZnO (*x* = 1, 2, 3, and 4).

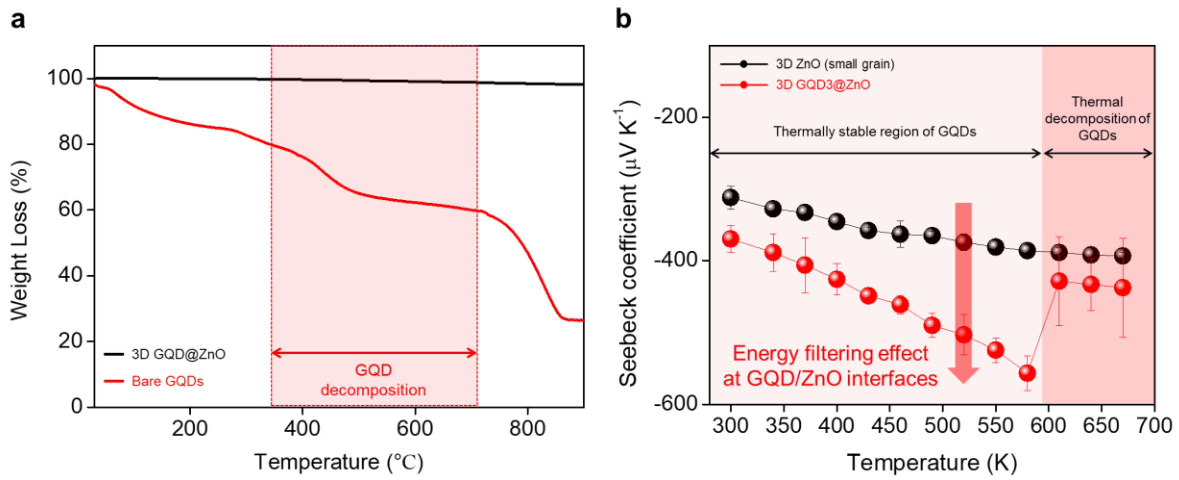

Supplementary Figure 13| Thermal stability of GQDs. a, Thermal gravimetric analysis (TGA) results of bare GQDs and 3D GQD@ZnO. **b,** Seebeck coefficient of 3D ZnO (small grain) and 3D GQD*x*@ZnO ($x = 3$) within the temperature range of 300-670 K. Error bars shown in the **Supplementary Fig. 13** represents the SD.

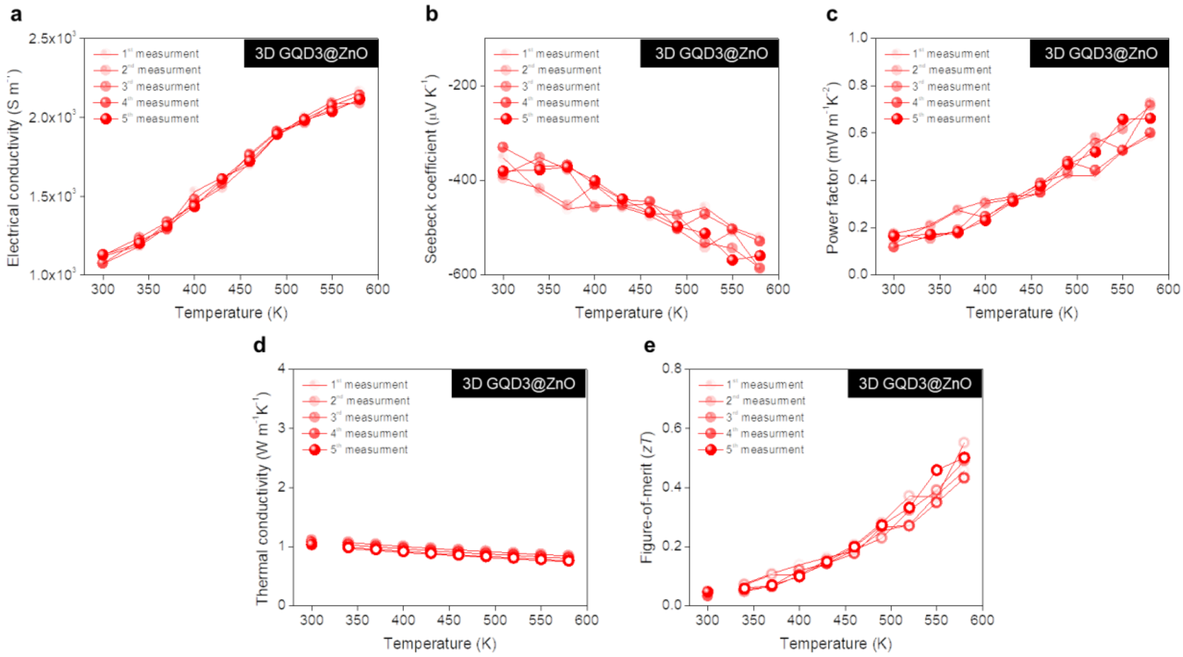

Supplementary Figure 14| Five sets of measurements for the thermoelectric properties of 3D GQD@ZnO. Temperature dependent (**a**) Seebeck coefficient, (**b**) electrical conductivity, (**c**) power factor, (**d**) thermal conductivity, and (**e**) zT values of 3D GQDx@ZnO (x = 3).

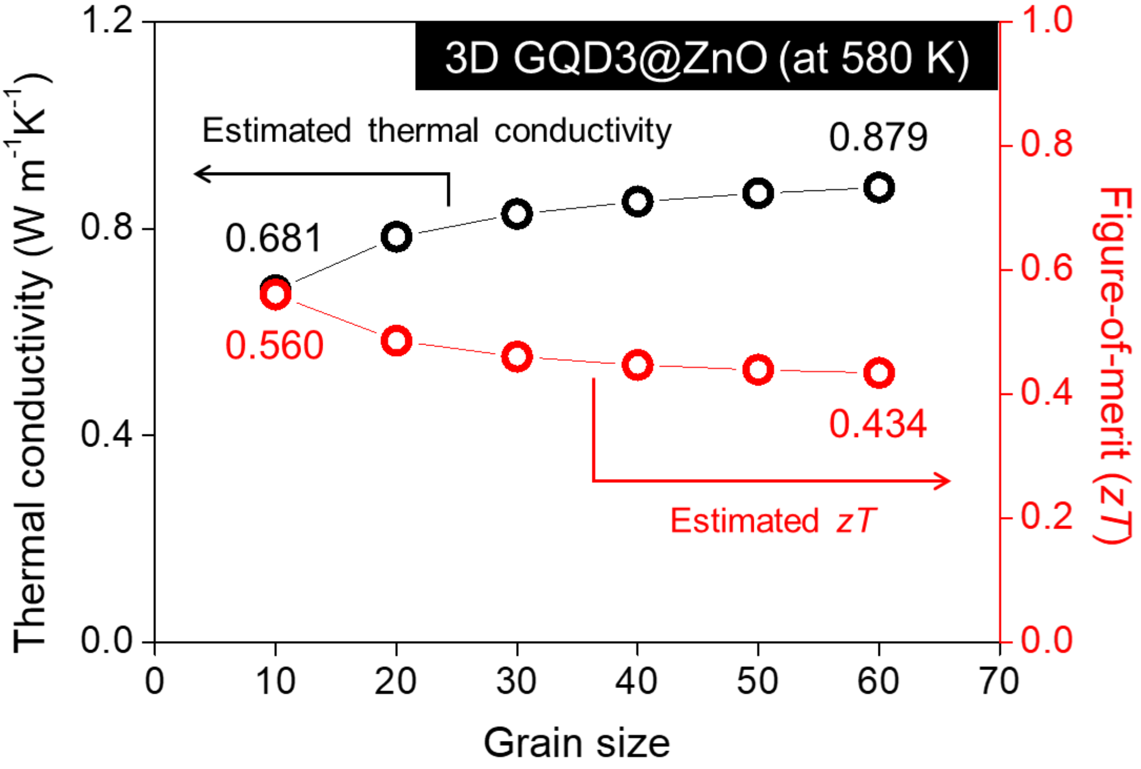

Supplementary Figure 15| Estimated thermal conductivity and *zT* values at 580 K for 3D GQD*x*@ZnO (*x* = 3) as a function of grain size for ZnO.

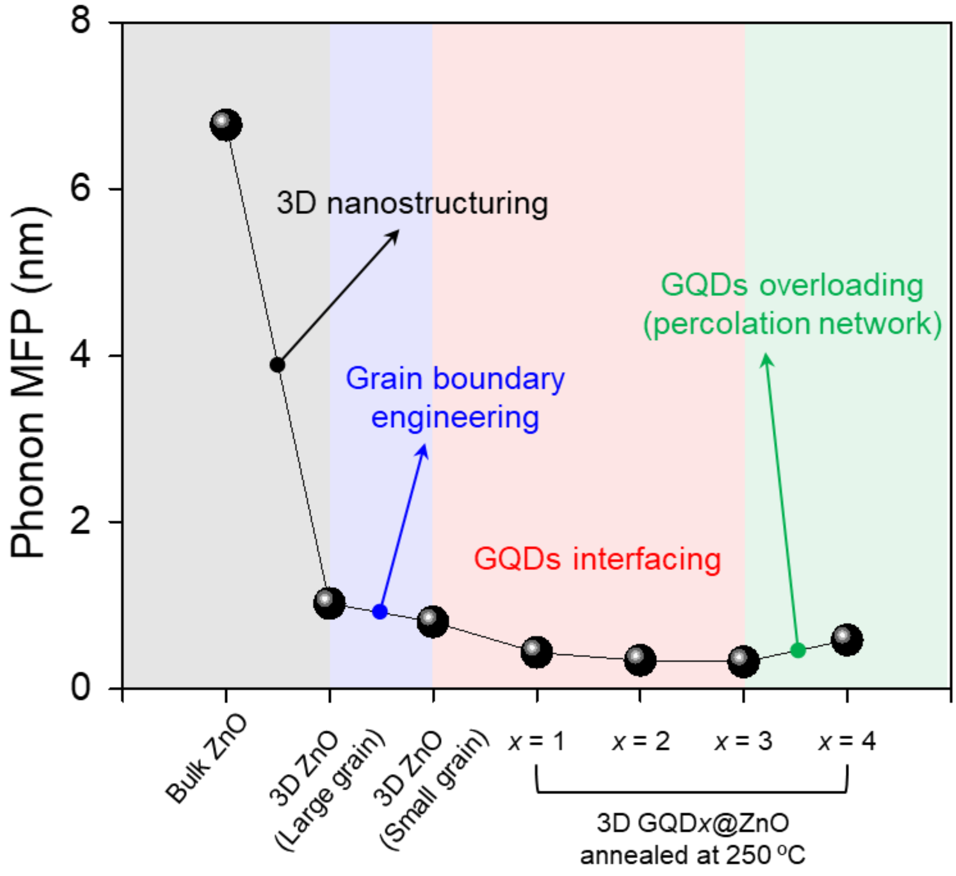

Supplementary Figure 16| Phonon mean free path (MFP) for bulk ZnO, 3D ZnO with different grain sizes, and 3D GQD*x*@ZnO (*x* = 1, 2, 3, and 4) at 300 K. Phonon MFP is calculated using kinetic theory based on the equation $k_{latt} = \frac{1}{3}C_v vl$, where k_{latt} is the lattice thermal conductivity, C_v is the specific heat capacity under a constant volume, v is the group velocity of phonons, and l is the phonon MFP.

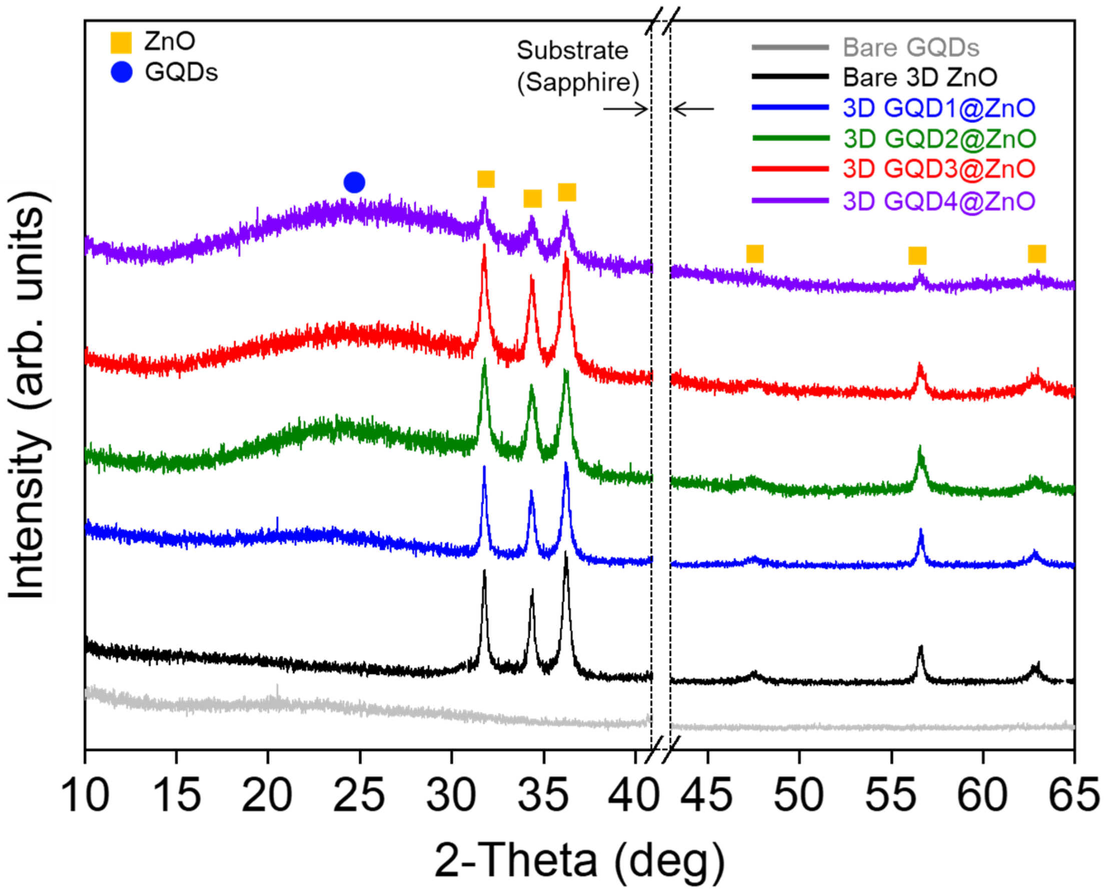

Supplementary Figure 17| XRD spectra of 3D ZnO and 3D GQD*x*@ZnO (x = 1, 2, 3, and 4).

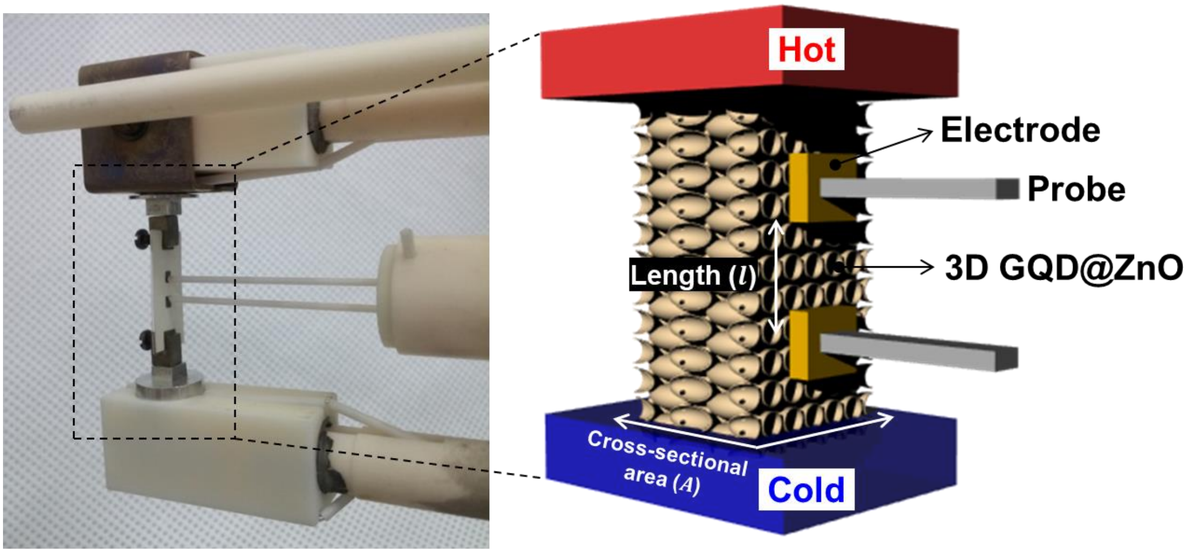

Supplementary Figure 18| Measurement system of electrical conductivity for 3D GQD@ZnO.

Supplementary Tables

Types	Materials	Figure-of-merit (zT)	T (K)	Power factor ($S^2\sigma$) ($\mu W\ cm^{-1}\ K^{-2}$)	Thermal conductivity (κ) ($W\ m^{-1}\ K^{-1}$)	Ref.
$SrTiO_3$	$Sr_{0.93}La_{0.07}Ti_{0.93}Nb_{0.07}O_3$	0.43	1009	12.6	~3.0	1
	$SrTi_{0.85}Nb_{0.15}O_3$	0.4	1100	11	3.2	2
	$Sr_{0.9}La_{0.067}TiO_3$/0.6 wt% graphene	0.36	1023	7.5	2.1	3
In_2O_3	$In_{1.88}V_{0.12}\ O_3$	0.42	973	7.81	~1.5	4
	$In_{1.98}Co_{0.02}O_3$	0.26	1073	4.6	1.8	5
	$In_{1.99}Ge_{0.01}O_3$	0.18	1000	-	3.6	6
$Ca_3Co_4O_9$	Sr-doped $Ca_3Co_4O_9$	0.29	1073	11.6	4.4	7
	$Ca_3Co_4O_9$ from $Co^{II}Co^{III}$-LDH	0.18	1000	3.1	1.6	8
$CaMnO_3$	Ca0.9Dy0.1MnO3	0.2	1000	3.56	~1.85	9
	Ca0.9Yb0.1MnO3	0.2	1000	~3	1.6	9
$Na_xCo_2O_4$	$Na_xCo_2O_4$/Ag composites	~0.5	~970	~1.4	~2.75	10
	$Na_xCo_2O_4$/Au composites	~0.44	~970	~1.2	~2.8	10

Supplementary Table 1| Thermoelectric properties of various metal oxides in previous studies.

Sample names	Seebeck coefficient (S) (S_{300K} (S_{580K}))	Electrical conductivity (σ) (σ_{300K} (σ_{580K}))	Thermal conductivity (κ) (k_{300K} (k_{580K}))	Figure-of-merit (zT) (zT_{300K} (zT_{580K}))
3D ZnO (Before annealing)	– 331 μV K^{-1} (– 388 μV K^{-1})	82 S m^{-1} (1540 S m^{-1})	2.13 W m^{-1}K^{-1} (1.21 W m^{-1}K^{-1})	0.001 (0.112)
3D ZnO (250 ℃, Small grain)	– 311 μV K^{-1} (– 385 μV K^{-1})	1416 S m^{-1} (2531 S m^{-1})	2.61 W m^{-1}K^{-1} (1.49 W m^{-1}K^{-1})	0.016 (0.147)
3D ZnO (500 ℃, Large grain)	– 269 μV K^{-1} (– 338 μV K^{-1})	2196 S m^{-1} (3154 S m^{-1})	3.32 W m^{-1}K^{-1} (1.90 W m^{-1}K^{-1})	0.014 (0.110)

Supplementary Table 2| Thermoelectric parameters of 3D ZnO with varying grain sizes.

Sample names	Seebeck coefficient (S) (S_{300K} (S_{580K}))	Electrical conductivity (σ) (σ_{300K} (σ_{580K}))	Thermal conductivity (κ) (k_{300K} (k_{580K}))	Figure-of-merit (zT) (zT_{300K} (zT_{580K}))
3D GQD1@ZnO	– 344 μV K^{-1} (– 446 μV K^{-1})	1199 S m^{-1} (2228 S m^{-1})	1.42 W $m^{-1}K^{-1}$ (0.979 W $m^{-1}K^{-1}$)	0.030 (0.263)
3D GQD2@ZnO	– 351μV K^{-1} (– 502 μV K^{-1})	1172 S m^{-1} (2181 S m^{-1})	1.11 W $m^{-1}K^{-1}$ (0.785 W $m^{-1}K^{-1}$)	0.039 (0.407)
3D GQD3@ZnO	– 369 μV K^{-1} (– 556 μV K^{-1})	1102 S m^{-1} (2123 S m^{-1})	1.05 W $m^{-1}K^{-1}$ (0.785 W $m^{-1}K^{-1}$)	0.043 (0.486)
3D GQD4@ZnO	– 276 μV K^{-1} (– 363 μV K^{-1})	1674 S m^{-1} (2794 S m^{-1})	1.90 W $m^{-1}K^{-1}$ (1.40 W $m^{-1}K^{-1}$)	0.020 (0.153)

Supplementary Table 3| Thermoelectric parameters of 3D GQD*x*@ZnO ($x = 1, 2, 3,$ and 4).

Symbols	Description	Value
υ	Average sound velocity	3100 m/s
d	Average grain size	0.02 μm
N_p	Number density of nanoprecipitates	6.4×10^{23} m^{-3} (GQD1)<br>1.28×10^{24} m^{-3} (GQD2)<br>1.32×10^{24} m^{-3} (GQD3, 4)
C	Average radius of nanoprecipitates	4 nm
θ	Debye temperature	400 K
P	Film porosity	0.793

Supplementary Table 4| Materials parameters used to calculate the lattice thermal conductivity.

Supplementary References

1. Li, Jian-Bo, *et al.* Broadening the temperature range for high thermoelectric performance of bulk polycrystalline strontium titanate by controlling the electronic transport properties. *J. Mater. Chem. C.* **6**, 7594-7603 (2018)

2. Zhang, Boyu, *et al.* High thermoelectric performance of Nb-doped $SrTiO_3$ bulk materials with different doping levels. *J. Mater. Chem. C.* **3**, 11406-11411 (2015)

3. Lin, Yue, *et al.* Thermoelectric power generation from lanthanum strontium titanium oxide at room temperature through the addition of graphene. *ACS Appl. Mater. Interfaces.* **7**, 15898-15908 (2015)

4. Ahmad, Abid, *et al.* Thermoelectric performance enhancement of vanadium doped n-type In_2O_3 ceramics via carrier engineering and phonon suppression. *ACS Appl. Energy Mater.* **3**, 1552-1558 (2019)

5. Liu, Yong, *et al.* Effect of transition-metal cobalt doping on the thermoelectric performance of In_2O_3 ceramics. *Am. Ceram. Soc.* **93**, 2938-2941 (2010)

6. Combe, Emmanuel, *et al.* Microwave sintering of Ge-doped In_2O_3 thermoelectric ceramics prepared by slip casting process. *J. Eur. Ceram. Soc.* **35**, 145-151 (2015)

7. Torres, M. A., *et al.* Significant enhancement of the thermoelectric performance in $Ca_3Co_4O_9$ thermoelectric materials through combined strontium substitution and hot-pressing process. *J. Eur. Ceram. Soc.* **39**, 1186-1192 (2019)

8. Delorme, Fabian, *et al.* Synthesis of thermoelectric $Ca_3Co_4O_9$ ceramics with high ZT values from a $Co^{II}Co^{III}$-Layered Double Hydroxide precursor. *Mater. Res. Bull.* **47**, 3287-3291 (2012)

9. Wang, Yang, Yu Sui, and Wenhui Su. High temperature thermoelectric characteristics of $Ca_{0.9}R_{0.1}MnO_3$ (R= La, Pr,…, Yb). *J. Appl. Phys.* **104** (2008)

10. Ito, Mikio, and Daisuke Furumoto. Effects of noble metal addition on microstructure and thermoelectric properties of $Na_xCo_2O_4$. *J. Alloys Compd.* **450**, 494-498 (2008)

- End of Supplementary Information -
